# Editorial

**Published:** 1867-03

**Authors:** 


### Editorial.

Injustice to the Insane.—We see in the recent biennial report of the Superintendent of the Hospital for the Insane at Jacksonville. Some pretty severe comments on the laws of this State relating to the method of proving persons insane, preparatory to admitting them into the Hospital. The comments of Dr. McFarland are not more severe, however, than they are just and well timed. The law positively requires the insane person to be brought into the Probate Court-room, before a jury, one of whom must be a physician, and such jury, after hearing the evidence, is to render a decision, as to whether the person present is sane or insane. Every intelligent physician knows that a large number of cases of insanity are of such a nature, that the public exhibition required by the law is directly calculated to aggravate the disease, sometimes even to a fatal extent. And yet the examination before the jury is practically a farce, because five out of six of the jurymen are entirely ignorant of the nature of insanity, and are obliged to rest their decision on the testimony of competent medical witnesses. Hence, the certificates of these same witnesses, duly sworn to, and handed directly to the judge, would afford precisely the same evidence of sanity or insanity, and just as much safety against abuses, as is afforded by the present law. The idea of bringing an insane person into a public court, for the purpose of testing the question of their insanity simply, is a barbarous absurdity.

Delegates to the International Medical Congress in Paris.—We learn that the New York State Medical Society, at its recent annual meeting, appointed several delegates to represent that Society in the International Medical Congress, to be held in Paris next August. We hope all the more important medical societies in this country will do the same, taking care, however, to send only men of eminent ability.

---

Chicago Medical Society.—The discussion at the last meeting of this Society, was on the entozoæ found in the human species, and their pathological effects. The discussion was an interesting one, and participated in by Drs. Wickersham, C. G. Smith, E. L. Holmes, Paoli, Groesbeck, Reed, Ross, and Davis. Nothing positively new was elicited, except a partial account of a very singular case now under observation in this city; and of which we hope to have a more complete report hereafter.

---

The Summer Course of Instruction.—The regular reading and Clinical Term of instruction in the Chicago Medical College will commence on the second Tuesday in March, and continue until the first of July following. The instruction will be given by the Faculty of the College, and will consist of one or two familiar lectures and recitations, and one hour of clinical instruction in the Hospitals each day. The clinics in the Mercy Hospital will be given on Monday, Wednesday, Thursday, and Saturday; and in the County and City Hospital, on Tuesday and Friday of each week.

Good *material* will also be furnished to those who wish to pursue dissections. The Spring and Summer Course, as thus arranged, affords very superior advantages to all such students as can remain in the City until July. It ensures a systematic course of practical and demonstrative instruction of the most valuable character, without the crowding of many topics upon the mind the same day, as is necessarily done during the regular Winter Term. It must be distinctly understood, however, that this Summer Reading and Clinical Term is merely supplimentary to the Annual Lecture Term, and in no case to be counted as *one college term* by the student in making up his requirements for graduation.

It has been no part of the objects of the Faculty of the Chicago Medical College to increase the *facilities* for the graduation of students, by making two Lecture Terms in one year, or by adhering to one short and inadequate term. On the contrary, one of their primary and most cherished objects, is to induce medical students to take a higher and broader course of medical instruction, before attempting to graduate. The Summer Course is free to all the matriculants of the College.

---

### PROCEEDINGS OF MORGAN COUNTY MEDICAL SOCIETY.

The Society met at the court house in Jacksonville, at 2 o'clock P.M., on Thursday the 14th day of February.

In the absence of the President and Vice-President Dr. Askew was appointed President *pro tem.*

The minutes of the last meeting were read and approved as printed in the JOURNAL. Drs. DeLeuw and C. A. Edgar were nominated for membership.

As no one of the individuals regularly appointed for the exercises of the meeting were prepared with papers, Dr. Malone was requested to read a paper on the Calabar Bean.

After giving its natural history, he proceeded to its action upon animals and upon the human system—when administered in the usual way—by subcutaneous injection and in other ways. Its local influence upon the eye was more particularly commented upon, and the experiments of quite a number of foreign scientific men were mentioned, and the results of those experiments were, briefly, that the local action of this drug produced first, a sensation of "strong tension in the neighborhood of the ciliary body," and second, "astigmatism or a state of irregular refraction in the eye in which the rays are not brought to one focus, but converge at different distances so as to form two linear images at right angles to each other." This peculiar condition of vision was remedied by concave glasses of proper foci. This astigmatic state existed eighteen hours after its application. Numerous experiments have proven that it invariably causes prompt contraction of the pupil, and that the contraction reaches its maximum in the course of an hour.

Dr. Malone was requested by the Society to publish his paper in some medical journal.

Dr. Prince had prepared and read "the chief points in a case of forward dislocation of the tibia on the astragalus with fracture of fibula, in which at the end of nearly three months the dislocation was found still to exist, having either never been reduced or having become re-dislocated. The displacement at the end of this period was reduced and the parts kept in position by an outside splint of wood, pasteboard, and bandage. The result was a prosecution for damages against the physician first in attendance, on account of alleged malpractice (tried three years after the receipt of the injury), the jury finding for the defendant.

The doctor read the instructions given by the judge to the jury in this case.

Dr. Edgar remarked that he was much interested in the case. The suit evidently grew out of malice. The man was hunted down by his medical brethren of the same county. It arose from want of professional courtesy.

The doctor hoped that medical men would rise above their petty feelings of jealousy and rivalry, and cease to give cause to the members of other professions to hold them upas proverbially quarrelsome.

Dr. Edgar, having prepared some notes on the early history of the introduction of mercurials into the materia medica, was requested to read them to the Society, which he accordingly did. They were listened to with marked attention and interest by the Society.

Dr. Reed made some remarks upon the use of mercurials.

The Society then adjourned to meet on the second Thursday in March, at half-past 1 o'clock P.M., at the court house, in Jacksonville.

C. T. WILBUR, M.D., *Secretary*.

---

The Body of Jeremy Bentham.—The London *Notes and Queries* contains a letter from the late Dr. Southwood Smith, in relation to the disposal of the body of Jeremy Bentham. The letter is dated June 14th, 1857, and says:—"Jeremy Bentham left by will his body to me for dissection. I was also to deliver a public lecture over his body to medical students and the public generally. The latter was done at the Webb Street School—Brougham, James Mill, Grote, and many other disciples of Bentham being present. After the usual anatomical demonstrations over the body, a skeleton was made of the bones. I endeavored to preserve the head untouched, merely drawing away the fluids by placing it under an air-pump over sulphuric acid. By this means the head was rendered as hard as the skulls of the New Zealanders, but all expression was gone, of course. Seeing this would not do for exhibition, I had a model made in wax by a distinguished French artist, taken from David's bust, Pickersgill's picture and my own ring. The artist succeeded in producing one of the most admirable likenesses ever seen. I then had the skeleton stuffed out to fit Bentham's own clothes, and this wax likeness fitted to the trunk. This figure was placed seated on the chair on which he usually sat, and one hand holding the walking stick which was his constant companion when he went out, called by him Dapple. The whole was enclosed in a mahogany case, with folding glass doors. When I removed from Finsbury Square I had no room large enough to hold the case; I therefore gave it to University College, where it now is.

———

A Novel Method for the Production of Anatomical Plates.—At a late meeting of the Vienna Academy of Sciences, Herr Mach suggested a curious application of photography to the purposes of anatomical education. "If," he said, "you photograph any solid body, such as a sphere, and if before the image has been fully received you substitute for the sphere another solid body, such as a cone, the resulting photograph will be a sort of transparent picture of a sphere enclosing a cone, which, when arranged stereoscopically, will seem perfectly solid and transparent." His application of this principle was thus expressed:—"Place a temporal bone before the camera, and before its image has been fully formed, substitute for it a cast of the auditory apparatus, and you will have a picture in which the internal ear can be seen through a transparent picture of the temporal bone. When this is arranged for the stereoscope, the two parts will be seen in perfect relief."

MONEY RECEIPTS FOR EXAMINER, FROM JAN. 1ST TO FEB. 21ST.—Drs. C. B. Reed $3, L. H. Cary 1, B. Stuvi 3, Wm. H. Byford 3, Orin Peak 3, V. C. Price 3, R. Winton 3, A. G. Jones 3, N. Holton 6, T. Scharer 3, C. M. Robertson 2, W. H. Lyford 3, J. W. Johns 3, J. McLaughlin 3, W. A. Elder 3, Philip Schaffer 3.50, J. S. Bullock 1, J. N. Bishop 3, H. Nance 3, V. H. Coffman 5, T. D. Fitch 5, J. M. Barlow 3, M. F. Bassett 3, Dr. Frazey 3, G. W. Morrill 2, T. Hoffman 3, H. W. Kendall 3, J. Brown 3, S. R. Millard 3, D. W. Young 10, T. D. Fisher 3, J. E. Thayer 2, J. Prestman 2, W. P. Naramore 3, S. B. Davis 5, A. Sheffield 3, Geo. Fredigke 3, Addison Niles 3, M. Shephard 3, J. F. McCormick 3, J. H. Goodell 3, T. M. Tyler 3, H. P. Oggell 3, C. T. Lichtenberger 5, M. Parker 3, A. M. Maxwell 3, E. P. Cook 3, A. G. Edwards 3, David Newell 3, L. Humphreys 3, R. Bolton 3.

